# Arc Characteristics of Ultrasonic-Magnetic Coaxial Hybrid GTAW

**DOI:** 10.3390/ma15228130

**Published:** 2022-11-16

**Authors:** Wenbo Du, Wenlong Li, Yue Li, Chao Chen

**Affiliations:** 1 National Key Laboratory for Remanufacturing, Army Academy of Armored Forces, Beijing 100072, China; 2 College of Mechanical and Electrical Engineering, Northeast Forestry University, Harbin 150040, China; 3 Beijing Institute of Radio Measurement, Beijing 100039, China

**Keywords:** ultrasonic, magnetic, GTAW, arc characteristics, orthogonal experiments

## Abstract

Ultrasonic-magnetic field coaxial hybrid GTAW(U-M-GTAW) is a new non-melting electrode welding method proposed by combining ultrasonic assisted GTAW(U-GTAW) and magnetic assisted GTAW(M-GTAW) on the regulation characteristics of the GTAW arc. U-M-GTAW introduces ultrasonic and magnetic field effects into GTAW to improve arc characteristics. The orthogonal experiment was designed to investigate the degree of influence of different process parameters on the arc. The degree of influence of ultrasonic power *P*, radiator height *H*, magnetic field current *C_W_*, welding current *C_W_* and tungsten electrode height *H_T_* on Δ*L*_1_ (degree of arc root diameter change), Δ*L*_2_ (degree of maximum diameter change) and Δ*S* (degree of area change) were analyzed. In the parameter range, *P* has the greatest degree of influence on Δ*L*_1_ and Δ*L*_2_. As all process parameters increase, *L*_1_ shows a tendency to decrease, indicating an increase in the compression of the arc root. Δ*L*_2_ with the increase in *P* and *C_W_* shows a trend of first decreasing and then increasing. Δ*L*_2_ with the increase in *H* decreases, indicating that the acoustic radiation force increases, the arc energy increases, and the dark region decreases. The magnetic field current increases, the bottom of the arc expands, and the height of the tungsten electrode increases, the arc dispersion and thus the difference between the dark and luminous regions at the bottom increases, resulting in Δ*L*_2_ with the increase in *C_M_* and H_T_ increases. *C_W_* has the greatest degree of influence on Δ*S*. Δ*S* decreases and then increases as *P* and *H* increase, which indicates that the force on acoustic radiation increases and then decreases in the range. An increase in the magnetic field current increases the rotation of the arc, leading to an increase in the arc area. An increase in welding current leads to an increase in arc energy, expansion of the arc morphology, and an increase in Δ*S*. The tungsten electrode height increases, the arc diverges, the dark region increases, the luminous area decreases, and Δ*S* increases. Finally, combined with the analysis of ultrasonic field and magnetic field theory, changes in process parameters will affect the force of the arc and thus the arc morphology. The U-M-GTAW arc under the action of acoustic radiation force, the plasma flow is shifted in the direction of the arc axis, and the arc contraction, under the action of magnetic field force to generate circumferential current, the arc undergoes periodic rotation, which improves GTAW arc characteristics.

## 1. Introduction

Gas tungsten arc welding (GTAW) is suitable for high-quality welding, with the advantages of stable arc, easy control of the heat source, excellent forming, and high quality of the weld bead, but its shortcomings such as shallow welding depth and low productivity limit the development of the method. To improve the welding productivity and expand the application range of GTAW, domestic and international scholars have carried out a lot of work to regulate the arc and improve the energy density of the arc, including plasma arc welding (PAW) [1], activating flux TIG (A-TIG) [2], multi-tungsten electrode TIG (A-TIG) [2], and keyhole TIG (K-TIG) [3].

Meanwhile, some scholars proposed a multi-field hybrid GTAW method to regulate the arc, such as U-GTAW [4] and M-GTAW [5]. Composite welding can effectively improve the energy utilization of the heat source, increase the welding speed, increase the weld penetration, and improve the quality of the weld bead, which is the future trend of GTAW process development. Ultrasound refers to the vibration frequency of more than 20 kHz sound waves, ultrasound is a carrier of energy transfer and energy form, when its intensity exceeds a certain value, it can make some physical, chemical, and biological properties or state of the sound-transmitting medium change through its interaction with the sound-transmitting medium, or to speed up the process of such changes, which is called power ultrasound [6]. U-GTAW is the introduction of power ultrasound with the welding arc as the medium to realize the compound of ultrasound energy and welding arc. When the ultrasound passes through the arc plasma, as the power ultrasound is applied energy, it will inevitably have a certain effect on the arc and the molten pool. Sun et al. [4,7,8,9,10] were the first to compound ultrasonic to arc. They found that after applying ultrasonic, the arc was compressed so that the pressure and temperature in its central region increased significantly, and the stiffness of the arc increased. At the same time, the pressure distribution of the arc obeyed a Gaussian distribution. Xie et al. [11] found that the ultrasonic composite arc under the joint action of the internal and external acoustic fields, the degree of plasma constraint is significantly increased, the brightness of the arc is enhanced, and the range of the high-temperature region of the arc column is extended to the anode, and the intermediate particles appear to agglomerate and jitter up and down at a certain frequency. Cai et al. [12] studied periodic ultrasonic assisted TIG welding of 2219 aluminum alloy and found that the coarse precipitation phase tended to be uniformly dispersed in the weld zone under ultrasonic vibration. Compared to TIG, the hardness of the weld zone increased by 8.43% and the tensile strength increased by 29.02%.

The research on the effect of magnetic fields on the welding process originated in the 1960s and was initially used for grain refinement [13]. Later, various scholars found that the magnetic field can change the arc morphology, influence the metal transfer process, control the heat input and molten pool behavior, improve the weld formation, joint organization, mechanical properties and physical and chemical properties, reduce welding defects and improve the quality of weld formation [14,15,16]. Fan et al. [17] modeled the arc and the melt pool and found that the maximum arc velocity as well as the melt pool velocity could be increased by the applied axial magnetic field. Liu et al. [18] found in axial magnetic field-assisted CMT welding of pure copper and aluminum alloys that the magnetic field reduced the average heat input and the thickness of the Al_2_Cu intermetallic compound layer formed at the interface from 50 µm to 5 µm. The joint strength increased with increasing magnetic field. For a 21 mT and 10 Hz magnetic field, the maximum loading force reached 1.67 kN, resulting in a 33% increase in joint strength. Li et al. [19] applied an axial magnetic field to the brazing of AA6061 aluminum alloy with HSLA350 steel, which found that the Lorentz force caused the aluminum matrix and the second term to be refined, and the intermetallic compounds at the weld interface were significantly reduced making the hardness of the joint increased by 14%. Anderson et al. [20] found that when TIG welding 304 stainless steel the applied magnetic field increased the weld area by 100%, narrowed the width of the fusion zone by 25% and increased the depth of fusion by 3.0 mm. Moreover, the external magnetic field reduces the percentage of δ ferrite, improves the filling of the weld metal to the melt pool, and reduces the primary and secondary dendrite spacing.

The previous research has confirmed the feasibility of U-M-GTAW. Compared with the normal welding arc, the composite arc has certain ultrasonic and magnetic properties that are a feature of the system, and experiments have found that the composite arc is more concentrated under the action of ultrasonic and magnetic fields while generating periodic rotation [21,22]. However, the relationship between different process parameters and the arc and the mechanism of action is not clear, thus, it is necessary to study the degree of influence of different process parameters on the arc characteristics of U-M-GTAW.

## 2. Experimental Materials and Methods

**Figure 1:**
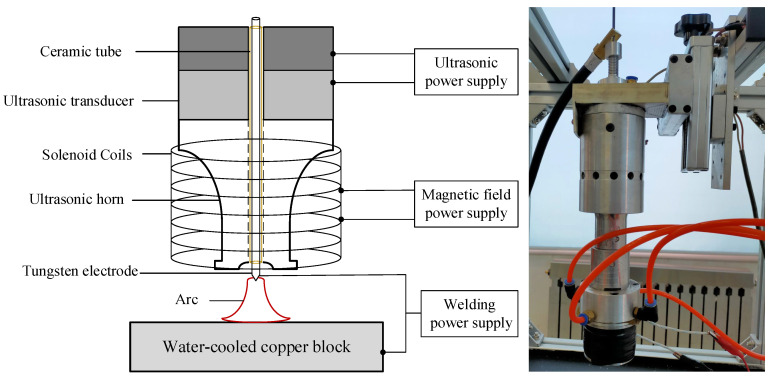
U-M-GTAW system.

The experiment was completed by U-M-GTAW system established based on the self-designed composite welding torch, whose structure is shown in Figure 1. The maximum output power of the ultrasonic power supply was 2400 W, and the vibration frequency was 20 kHz. The Master TIG 335AC/DC welding power supply was used to provide a stable welding process. The maximum output current of the magnetic field power supply was 3 A, the AC pulse frequency is 40 Hz, and the number of solenoid coil turns was 140. The protection flow was 25 L/min (99.99%Ar). The shooting frame rate of the high-speed camera was 2000 fps, and the exposure time was 20 μs. The tungsten electrode diameter was 3 mm, and the tip angle was 30°. The tungsten electrode tip loss was continuously observed during the experiment to ensure that the tungsten electrode angle remained unchanged, and the welding anode was a water-cooled copper block.

**Figure 2:**
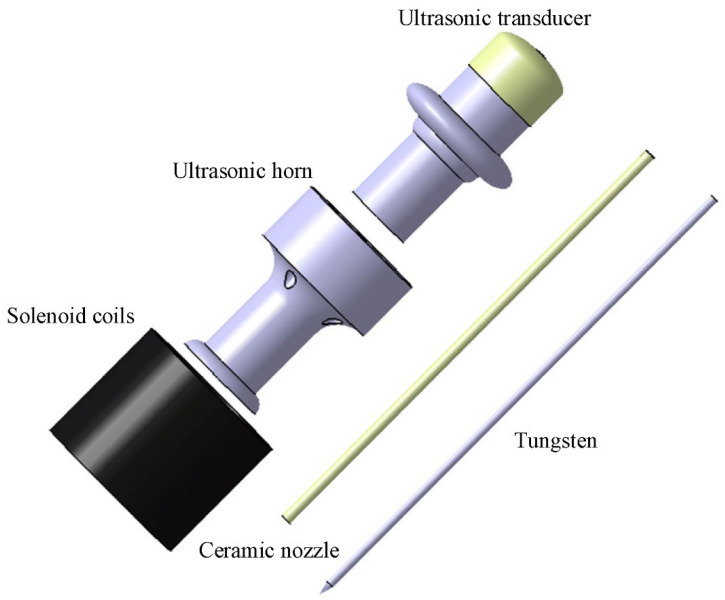
The exploded diagram of the composite welding torch.

The core component of the U-M-GTAW was the composite torch; which consisted of a modified common welding torch; ultrasonic transmitting equipment consisted of an ultrasonic transducer and an ultrasonic horn; and a solenoid coil. The exploded diagram of the composite welding torch is shown in Figure 2. Power ultrasound was generated by an ultrasonic transducer that converted the electrical signal output from the ultrasonic power supply into mechanical vibration, which was amplified by an ultrasonic horn and finally transmitted to the welding arc to achieve the impact of ultrasound on the welding process. The ceramic nozzle passed through the hollow through-hole of the ultrasonic transducer and the horn; and the tungsten electrode passed through the ceramic nozzle insulated from the ultrasonic emission equipment. The solenoid coils were rigidly connected to the ultrasonic horn; so that the ultrasonic emission equipment; tungsten electrode and solenoid coils were in the same axis by mechanical coupling. This structure could ensure the continuous transmission of ultrasonic energy and avoid the phenomenon of arc bias blowing during ultrasonic application; and also made the designed composite welding torch compact.

**Figure 3:**
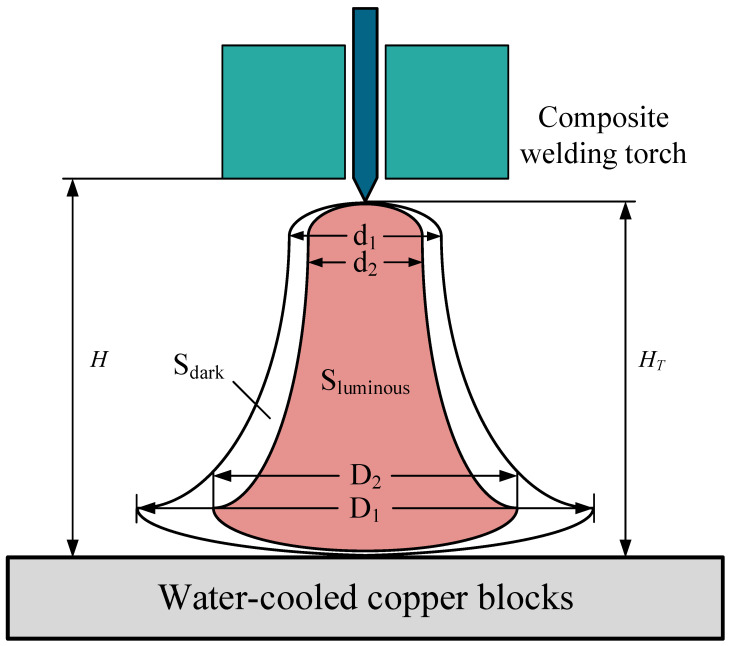
The arc parameter diagram.

Arc measurement parameters are shown in Figure 3: the dark region and luminous region are expressed in *S*_1_ and *S*_2_, respectively, the diameter of the dark region and luminous region at the root of the arc are *d*_1_ and *d*_2_, the diameters of the dark region and luminous region at the maximum diameter were *D*_1_ and *D*_2_. Using the first set of data as a baseline, the arc variation degrees at different locations were expressed as Δ*L*_1_ (Degree of arc root diameter change), Δ*L*_2_ (Degree of maximum diameter change), and Δ*S* (Degree of area change), as shown in Equations (1)–(3):

(1) $$\Delta L_{1}=\frac{d_{1}-d_{2}}{D_{2-1}}\times 100\%$$

(2) $$\Delta L_{2}=\frac{D_{1}-D_{2}}{D_{2-1}}\times 100\%$$

(3) $$\Delta S=\left|\frac{S_{1}-S_{2}}{S_{2-1}}\times 100\%\right|$$

**Figure 4:**
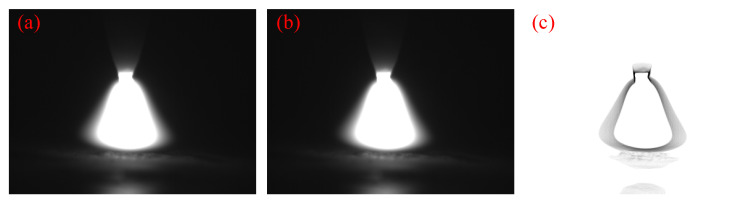
Arc image processing process. (**a**) original image, (**b**) threshold processing image, and (**c**) extracted image.

In order to better distinguish the boundary of the arc parameters, the arc image was processed as follows. Figure 4a shows the original image, the pixels below a certain threshold are removed from the image, as shown in Figure 4b, and then the brightness of the arc image is output as one color for the part above a certain value and another color for the part below a certain value, as shown in Figure 4c.

Orthogonal experiments were designed to better investigate the degree of influence of different factor variations on the arc morphology of U-M-GTAW, choosing ultrasonic power (*P*), radiator height (*H*), magnetic field current (*C_M_*), welding current (*C_W_*), and tungsten electrode height (*H_T_*) as the five factors, and three optimal parameters were selected as levels for each factor based on the results of previous single-factor experiments.

## 3. Results

**Figure 5:**
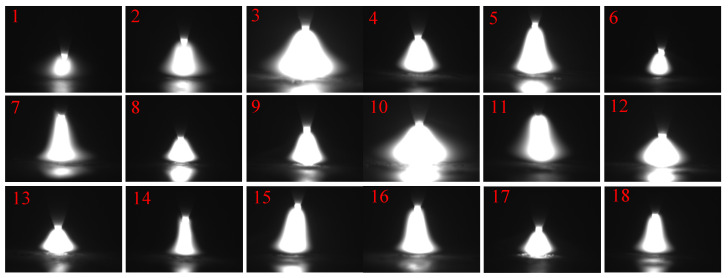
The arc images. (Note: the numbers 1,2,3,…..18 is expressed the images of arc shape in Table 1).

The orthogonal experimental table and results are shown in Table 1, and the arc images are shown in Figure 5.

**Figure 6:**
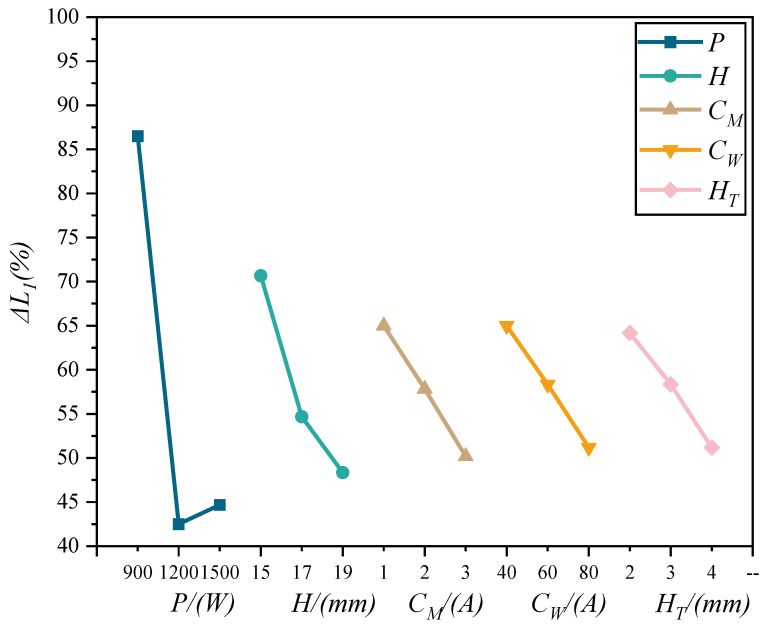
Trend diagram of Δ*L*_1_ with the variation of process parameters.

The results of the analysis of Δ*L*_1_ for different process parameters are shown in Table 2 and the trend diagram of the influence of process parameters on Δ*L*_1_ is shown in Figure 6.

As seen in Table 2, the range of *P* is much larger than that of other factors, indicating that *P* has the greatest degree of influence on Δ*L*_1_, and the degree of influence on Δ*L*_1_ is *P* > *H* > *C_W_* > *C_M_* > *H_T_*. From Figure 5, Δ*L*_1_ decreases and then slightly increases with increasing *P*, which indicates that the contraction of the arc root is better when *P* is at 1200 W and decreases with increasing *H*, *C_M_*, *C_W_*, and *H_T_* in the above parameter range. As *H* increases, the intensity of the standing wave ultrasonic field increases, the acoustic radiation force increases, the degree of arc contraction increases, and Δ*L*_1_ decreases. With the increase in *C_M_*, the compression effect of electromagnetic force on the root of the arc increases, and Δ*L*_1_ decreases. With the increase in *C_W_*, the arc energy increases, the dark bright area decreases, and Δ*L*_1_ decreases. With the increase in HT, the arc length increases, the radial diameter of the arc decreases, and Δ*L*_1_ decreases.

**Figure 7:**
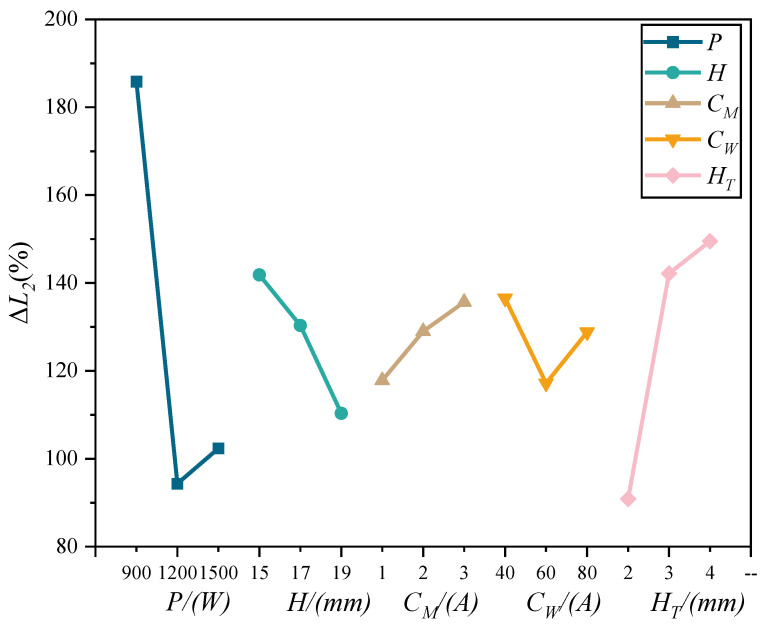
Trend diagram of Δ*L_2_* with the variation of process parameters.

The results of the analysis of Δ*L*_2_ for different process parameters are shown in Table 3 and the trend diagram of the influence of process parameters on Δ*L*_2_ is shown in Figure 7.

Figure 3 the degree of influence on Δ*L*_2_ is *P* > *H_T_* > *H* > *C_W_* > *C_M_*. In Figure 6, Δ*L*_2_ tends to decrease and then increase with the increase in *P* and *C_W_*, which indicates that the compression of Δ*L*_2_ is better when *P* = 1200 W and *C_W_* = 60 A. With the increase in *C_M_* and *H_T_* Δ*L*_2_ tends to increase, which indicates that the amplitude of arc rotation increases and the arc bottom expands. The decreasing trend with increasing *H* indicates that the acoustic radiation force increases and the arc contracts.

**Figure 8:**
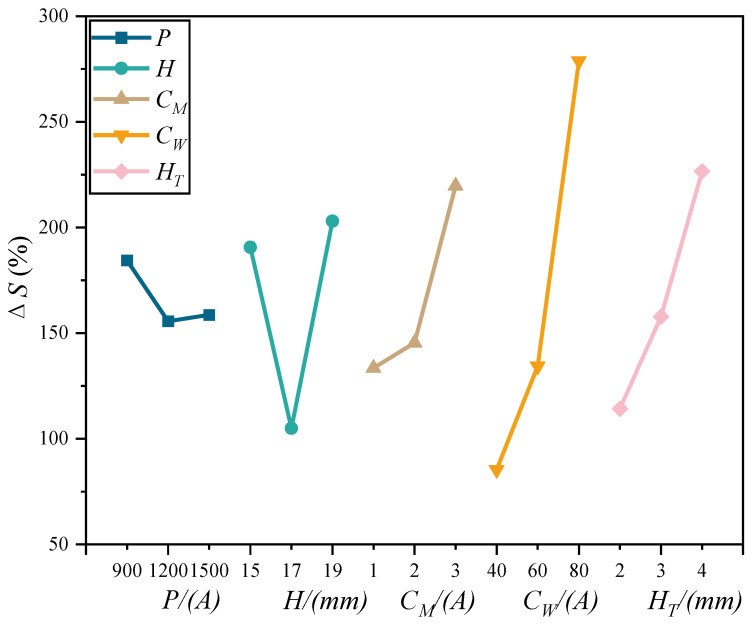
Trend diagram of Δ*S* with the variation of process parameters.

The results of the analysis of Δ*S* for different process parameters are shown in Table 4 and the trend diagram of the influence of process parameters on Δ*S* is shown in Figure 8.

From Table 4, the degree of influence on Δ*S* is *C_W_* > *H_T_* > *H* > *C_M_* > *P*. In Figure 7, Δ*S* increases with the increase in *C_M_*, *C_W,_* and *H_T_*, which is caused by the increase in *C_M_*, the increase in arc rotation amplitude so that the increase in arc area, the increase in arc energy with the increase in *C_W_*, the decrease in dark region, the increase in luminous region, and the increase in *H_T_*, the arc elongation, the expansion of luminous region downward, and the increase in area. Δ*S* increases. With the increase in *P* and *H*, Δ*S* first shows a trend of decreasing and then increasing, which means that the arc compression effect is better when *P* = 1200 W and *H* = 17 mm.

## 4. Discussions

Plasma is a collective term for charged and neutral particles in arc space, including cations, electrons, molecules and atoms. The motion behavior of the particles in the arc space is very complex, there is a violent collision between the particles, to achieve the exchange of arc heat, transfer process, and the regular movement of electrons can achieve the current conduction, and through the collision between the particles will transfer energy to the neutral particles and ionization, is the arc itself to create charged particles to maintain its conductivity of the most important way.

**Figure 9:**
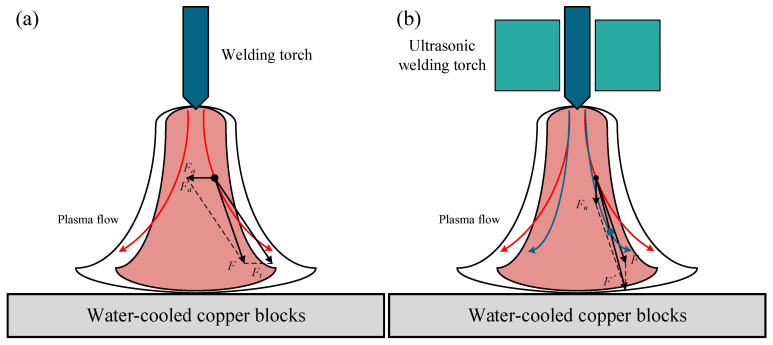
Arc force models. (**a**) traditional welding torch, (**b**) Ultrasonic welding torch.

The mechanical force of arc mainly includes electromagnetic contraction force and plasma flow force. When the current flows through the conductor, the entire current can be seen as several parallel current lines, these current lines will produce mutual attraction between the conductor cross-section tends to shrink. It will produce cross-sectional contraction, a phenomenon called electromagnetic contraction effect, and the resulting force is called electromagnetic contraction force. The plasma flow force in the arc is formed by the high-speed flow of high-temperature particles. The action of the arc static pressure causes a certain pressure difference between the arc cathode and the workpiece, resulting in a continuous flow, i.e., plasma flow. The additional pressure formed when it reaches the surface of the workpiece is called the plasma flow force. Among the arc forces, the plasma flow force is the main one and plays a decisive role, which can account for more than 80% of the total mechanical force of the arc. For the arc in the plasma flow at any position in a particle force analysis, as shown in Figure 9a.

*F_t_* is plasma flow force, *F_a_* is electromagnetic contraction force, whose magnitude is determined by Equations (4) and (5), respectively. Visible, electromagnetic contraction force and plasma flow force are both numerical quantities related to the arc current, arc distribution form, and form.

(4) $$F_{a}=KI^{2}\mathrm{lg}\left(R_{b}/R_{a}\right)$$

where *R_b_* is the radius of the bottom surface of the arc; *R_a_* is the radius of the root surface of the arc; *K* is the coefficient, *K = μ/4π* (*μ* is the magnetic permeability of the medium).

(5) $$F_{t}=(K_{1}/2)I^{2}$$

where *K*_1_ is the parameter related to the form of arc pressure distribution; *I* is the welding current.

The conventional GTAW arc is subjected to a combined electromagnetic force *F_a_* and plasma flow force *F_t_*, resulting in resultant force *F*. The resultant force *F* deflects farther from the electrode axis, resulting in an extension of the arc morphology. When the current increases, *F_a_* and *F_t_* increase, the combined force *F* increases, so the arc morphology increases, the degree of change in area increases. The arc heat production increases, the luminous area increases, so the degree of change in the diameter tends to decrease.

The acoustic radiation force *F_u_* generated by ultrasound is a single direction force along the direction of ultrasound propagation, i.e., along the axis of the arc, as shown in Figure 9b.

The acoustic radiation force *F_u_* together with the resultant force *F* of the plasma flow force and the electromagnetic contraction force produce a new resultant force $F^{\prime }$, which is more biased in the direction of the axis of the arc. The intervention of the acoustic radiation force *F_u_* changes the original flow direction of the plasma flow, forcing the arc particles to be displaced along the direction of the resultant force $F^{\prime }$, and the particle position moves off axis. Because the whole arc is in the ultrasonic field effect, all the particles in the arc are subjected to acoustic radiation force and forced displacement, the particles from the cathode to the anode trajectory shortened, which is manifested as the overall compression of the arc. The U-GTAW arc is strongly compressed in the radial direction due to the constraining effect of the additional acoustic radiation force, and the arc temperature, energy density, and plasma flow rate are significantly increased. In addition, the magnitude of the acoustic radiation force is influenced by the phase difference between the incident and reflected waves, and *H* is the most important factor affecting the phase difference. At some specific values of *H*, the incident ultrasonic waves and the reflected waves peak simultaneously, and the medium-fixed acoustic field is the resonant field with the maximum energy density. At some other specific values of *H*, the incident ultrasonic waves and the reflected waves cancel, which means that the amplitude of the standing waves is zero and the ultrasonic field has the lowest energy density. Therefore, the change in *H* affects the magnitude of the acoustic radiation force and thus the degree of change in the arc morphology.

Axial magnetic field distribution in the arc is the middle of the magnetic field density is higher, the surrounding magnetic field density is lower, and the greater the frequency of the magnetic field, the faster the speed of magnetic field change, the more uneven the distribution of magnetic lines of force inside and outside the arc, and charged particles in the axial magnetic field is the trajectory of the spiral curve, both radial motion, and axial motion [23]. When an axial magnetic field is applied in addition to the applied ultrasonic field, the plasma is then subjected to magnetic forces in addition to *F_a_*, *F_t_*, and *F_L_*.

The distance from any charged particle in the arc to the axis of the arc is *r*, and the current *I* at this point can be decomposed into the axial components *I_xy_* and *I_z_*, the angle between the current *I* and the axial component *I_z_* is θ, $I_{z}=I\cos\theta$, and $I_{xy}=I\sin\theta$ so that the Ampere’s force *F_A_* per unit arc column at the point on the arc is:

(6) $$F_{A}=BI_{xy}=BI\sin\theta$$

For the unit arc column length, it is subject to the aerodynamic drag is:

(7) $$F_{D}=\frac{1}{2}\rho v^{2}AC_{D}$$

where $F_{D}$ is aerodynamic drag; $C_{D}$ is aerodynamic drag coefficient; v is the speed of arc column motion; ρ is the gas density; A is the windward area per unit length of arc column.

When the unit particle reaches a steady state, i.e., when a circumferential current is generated within the arc, the arc rotates. The greater the magnetic field current, the greater the *F_A_*, the greater the circumferential current of the arc in the steady state, and therefore the greater the rotation of the arc, the greater the Δ*S*. It is found that in the arc length is long enough or the particle motion is slow in the arc, the Lorentz force *F_M_* on the particle in the upper middle section is greater than the centrifugal force *F_M_*’ of the current particle motion, the radius of particle motion gradually decreases, the arc shows contraction. In the middle and lower sections of the particle force is the opposite arc shows expansion. Therefore, Δ*L*_1_ and Δ*L*_2_ show a decreasing and increasing trend, respectively [5,24].

**Figure 10:**
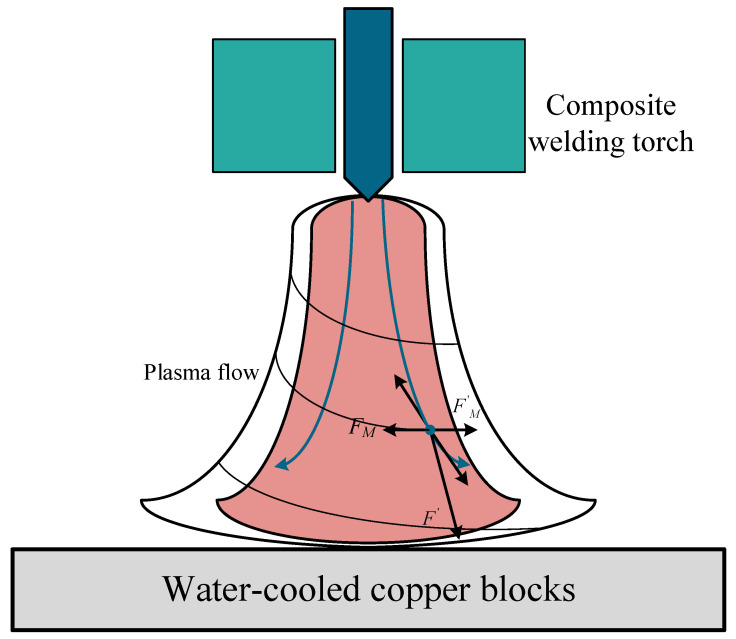
U-M-GTAW arc force mode.

At this point, the combined force with the magnetic field forces acting on the U-M-GTAW arc, as shown in Figure 10, resulting in the arc both contraction and periodic rotation of the arc characteristics. When the process parameters change, the force of the arc changes and affects the arc morphology.

## 5. Conclusions

1. The U-M-GTAW welding system was designed, and the experimental results showed that the U-M-GTAW arc rotates periodically while compressing under the action of the applied ultrasonic field and the axial magnetic field, which combines the effects of ultrasonic GTAW and magnetically controlled GTAW on the arc.
2. The orthogonal experiment was designed to investigate the effect of different process parameters on the degree of change in arc morphology, and the degree of effect on Δ*L*_1_ was *P* > *H* > *C_W_* > *C_M_* > *H*, the degree of effect on Δ*L*_2_ was *P* > *H_T_* > *H* > *C_W_* > *C_M_*, and the degree of effect on Δ*S* is *C_W_* > *H_T_* > *H* > *C_M_* > *P*.
3. Within the above parameters, Δ*L*_1_ decreases and then slightly increases with increasing *P*, and decreases with increasing *H*, *C_M_*, *C_W_*, and *H_T_*. Δ*L*_2_ tends to decrease and then increase with increasing *P* and *C_W_*, increases with increasing *C_M_* and *H_T_*, and decreases with increasing *H*. Δ*S* increases with increasing *C_M_*, *C_W_*, and *H_T_*. With the increase in *P* and *H*, Δ*S* tends to increase first and then decrease.
4. U-M-GTAW under the action of acoustic radiation force, the arc is strongly compressed in the radial direction, and the temperature, energy density, and plasma flow rate of the arc are significantly increased. Periodic rotation is generated under the action of the magnetic field force. When the process parameters change, the force of the arc changes and affects the arc morphology.

## Figures and Tables

**Table 1:** The orthogonal experimental table and results.

NO.	*P*/(W)	*H*/(mm)	*C_M_*/(A)	*C_W_*/(A)	*H_T_*/(mm)	Δ*L*_1_/(%)	Δ*L*_2_/(%)	Δ*S*/(%)
1	900	15	1	40	2	156	121	108.56
2	900	17	2	60	3	100	194	3.89
3	900	19	3	80	4	56	192	508.12
4	1200	15	1	60	3	43	105	172.20
5	1200	17	2	80	4	40	90	316.72
6	1200	19	3	40	2	34	67	59.63
7	1500	15	2	40	4	47	184	124.78
8	1500	17	3	60	2	41	73	107.18
9	1500	19	1	80	3	42	71	208.22
10	900	15	3	80	3	76	260	352.89
11	900	17	1	40	4	64	224	9.79
12	900	19	2	60	2	67	124	123.07
13	1200	15	2	80	2	48	79	154.88
14	1200	17	3	40	3	44	120	60.31
15	1200	19	1	60	4	46	105	169.93
16	1500	15	3	60	4	54	102	230.29
17	1500	17	1	80	2	39	81	132.09
18	1500	19	2	40	3	45	103	149.28

**Table 2:** Results of the orthogonal experimental analysis of Δ*L_1_*.

Mean	*P*/(W)	*H*/(mm)	*C_M_*/(A)	*C_W_*/(A)	*H_T_*/(mm)
Level 1/(%)	86.500	70.667	65.000	65.000	64.167
Level 2/(%)	42.500	54.667	54.833	58.500	58.333
Level 3/(%)	44.667	48.333	50.833	50.167	51.167
Range/(%)	44.000	22.334	14.167	14.833	13.000

**Table 3:** Results of the orthogonal experimental analysis of Δ*L*_2_.

Mean	*P*/(W)	*H*/(mm)	*C_M_*/(A)	*C_W_*/(A)	*H_T_*/(mm)
Level 1/(%)	185.833	141.833	117.833	136.500	90.833
Level 2/(%)	94.333	130.333	129.000	117.167	142.167
Level 3/(%)	102.333	110.333	135.667	128.833	149.500
Range/(%)	91.500	31.500	17.834	19.333	58.667

**Table 4:** Results of orthogonal experimental analysis of Δ*S*.

Mean	*P*/(W)	*H*/(mm)	*C_M_*/(A)	*C_W_*/(A)	*H_T_*/(mm)
Level 1/(%)	184.387	190.600	133.465	85.392	114.235
Level 2/(%)	155.612	104.997	145.437	134.427	157.798
Level 3/(%)	158.640	203.042	239.737	278.820	226.605
Range/(%)	28.775	98.045	86.272	193.428	112.370

## Data Availability

Not applicable.

## References

[1] Zhang C., Wu C.S., Tian S.S. (2020). Effect of ultrasonic vibration on current density and keyholing capability of plasma arc. Sci. Technol. Weld. Join..

[2] Huang B., Yang J., Yin W., Chen P., Zhu Y., Li J. (2016). Research Progress and Prospect of A-TIG Welding. Mater. Rev..

[3] Liu Z.M., Chen S.Y., Yuan X., Zuo A., Zhang T., Luo Z. (2018). Magnetic-enhanced keyhole TIG welding process. Int. J. Adv. Manuf. Technol..

[4] Sun Q., Lin S., Yang C., Fan Y. (2008). The arc characteristic of ultrasonic assisted TIG welding. China Weld..

[5] Chang Y., Yang X., Li D., Li D. (2010). Arc shapes of TIG welding in a longitudinal magnetic field. Trans. China Weld. Inst..

[6] Zhao J., Dai G., Han J., Huang X. (2012). Research Progress of Application of Power Ultrasound in Metal Wwelding. Hot Work. Technol..

[7] Sun Q.J., Lin S.B., Yang C.L., Zhao G.Q. (2009). Penetration increase of AISI 304 using ultrasonic assisted tungsten inert gas welding. Sci. Technol. Weld. Join..

[8] Yangyang F.A.N., Qingjie S.U.N., Chunli Y., Lin S.B. (2009). TIG welding of the stainless steel 304 based on the ultrasonic vibration. Trans. China Weld. Inst..

[9] Fan C., Yang C., Lin S., Zhang Y. (2013). Arc Characteristics of Ultrasonic Wave-Assisted GMAW. Weld. J..

[10] Sun Q. (2011). Characteristic of Arc Pressure in Ultrasonic-TIG Hybrid Welding. J. Mech. Eng..

[11] Xie W., Fan C., Yang C., Lin S., Zhang Y. (2015). Characteristics of acoustic-controlled arc in ultrasonic wave-assisted arc. Acta Phys. Sin..

[12] Cai X., Lin S., Wang X., Yang C., Fan C. (2019). Characteristics of Periodic Ultrasonic Assisted TIG Welding for 2219 Aluminum Alloys. Materials.

[13] Brown D.J.W.J. (1962). The Effect of Electromagnetic Stirring and Mechanical Vibration. Weld. J..

[14] Shoichi M., Yukio M., Koki T., Yasushi T., Yukinori M., Yusuke M. (2013). Study on the application for electromagnetic controlled molten pool welding process in overhead and flat position welding. Sci. Technol. Weld. Join..

[15] Chen J., Wei Y., Zhan X., Pan P. (2017). Weld profile, microstructure, and mechanical property of laser-welded butt joints of 5A06 Al alloy with static magnetic field support. Int. J. Adv. Manuf. Technol..

[16] Li M., Xu J., Huang Y., Rong Y. (2018). Improving Keyhole Stability by External Magnetic Field in Full Penetration Laser Welding. JOM.

[17] Xiao L., Fan D., Huang J. (2018). Tungsten cathode-arc plasma-weld pool interaction in the magnetically rotated or deflected gas tungsten arc welding configuration. J. Manuf. Process..

[18] Liu Y.B., Sun Q.J., Wang H., Zhang H.M., Cai S.J., Feng J.C. (2016). Effect of the axial external magnetic field on copper/aluminium arc weld joining. Sci. Technol. Weld. Join..

[19] Li R., Yuan X., Zhang H., Yang J., Wu K., Li T., Wang G., Tao S. (2021). Effect of axial magnetic field on TIG welding–brazing of AA6061 aluminum alloy to HSLA350 steel. J. Mater. Res. Technol..

[20] Queiroz A.V.D., Fernandes M.T., Silva L., Demarque R., Xavier C.R., De Castro J.A. (2020). Effects of an External Magnetic Field on the Microstructural and Mechanical Properties of the Fusion Zone in TIG Welding. Metals.

[21] Chen C., Li W., Du W., Du W., Liu J., Zhang H. (2022). Feasibility analysis of standing wave ultrasonic—Axial magnetic field hybrid for controlling GTAW arc characteristics. J. Manuf. Process..

[22] Chen C., Li W., Fan C., Du W., Zhang H. (2022). Understanding the changing mechanism of arc characteristics in ultrasound-magnetic field coaxial hybrid gas tungsten arc welding. Plasma Sci. Technol..

[23] Chen S., Meng D., Su Z., Jiang F., Lu Y. (2014). Effects of longitudinal magnetic field on non-consumable gas shielded arc welding. Trans. China Weld. Inst..

[24] Wu H., Chang Y.L., Babkin A., Lee B. (2020). The behavior of TIG welding arc in a high-frequency axial magnetic field. Weld. World.

